# Oxidative stress and antioxidant defense in patients with chronic hepatitis C patients before and after pegylated interferon alfa-2b plus ribavirin therapy

**DOI:** 10.1186/1479-5876-4-25

**Published:** 2006-06-20

**Authors:** Görenek Levent, Acar Ali, Aydın Ahmet, Eyigun Can Polat, Çetinkaya Aytaç, Eken Ayşe, Sayal Ahmet

**Affiliations:** 1 Gülhane Military Medical Academy, Department of Infectious Diseases and Clinic Microbiology, 06018, Etlik, Ankara, Turkey; 2 Gülhane Military Medical Academy, Department of Toxicology, Ankara, Turkey

## Abstract

**Background:**

Oxidative stress could play a role in pathogenesis of hepatitis C virus (HCV) infection. The aim of our study is to determine oxidant/antioxidant status of patients with chronic hepatitis C (CHC), and the effect of pegylated interferon alfa-2b plus ribavirin combination therapy on oxidative stress.

**Methods:**

Nineteen patients with chronic HCV infection and 28 healthy controls were included in the study. In control and patient groups, serum alanine aminotransferase (ALT) and aspartate aminotransferase (AST) levels, erythrocyte malondialdehyde (MDA) levels, erythrocyte CuZn-superoxide dismutase (SOD), erythrocyte glutathione peroxidase (GSH-Px) activities were measured. After pegylated interferon alfa-2b and ribavirin combination therapy for 48 weeks, these parameters were measured again in the patient group.

**Results:**

Serum MDA levels increased significantly in CHC patients (n:19), before the treatment when compared with healthy subjects (n:28) 9.28 ± 1.61, 4.20 ± 1.47 nmol/ml, *p *< 0.001 respectively. MDA concentration decreased significantly (*p *< 0.001) after the treatment as well as ALT, AST activity, in erythrocytes of these patients. Average antioxidant enzymes (superoxide dismutase and glutathione peroxidase) were significantly lower in erythrocytes of patients with CHC before treatment compared with the control group (both, p < 0.001). Chronic Hepatitis C patients after pegylated interferon alfa-2b and ribavirin therapy showed values of SOD, GSH-Px were significantly higher than pretreatment levels (both, p < 0.001).

**Conclusion:**

Our results show that patients with chronic HCV infection are under the influence of oxidative stress associated with lower levels of antioxidant enzymes. These impairments return to level of healthy controls after pegylated interferon alfa-2b plus ribavirin combination therapy of CHC patients. Although interferon and ribavirin are not antioxidants, their antiviral capacity might reduce viral load, and inflammation, and perhaps through this mechanism might reduce virus-induced oxidative stress.

## Background

Hepatitis C virus (HCV) is one of the main causative agents of chronic viral hepatitis. Chronic hepatitis C can progress to cirrhosis and eventually to hepatocellular carcinoma over a period of 20 to 30 years. The mechanisms by which HCV causes cell damage are not well understood. Different mechanisms including immunological liver damage, direct cytotoxicity mediated by different viral product and inductions of oxidative stress have been suggested as playing a pathogenic role in this infection [1]. It has been suggested that HCV may cause oxidative stress in infected cell. Several lines of evidence support this contention, including the existence of activated glutathione turnover, the presence of increased levels of lipid peroxidation products and augmented iron stores in the liver, and the finding of diminished reduced glutathione values in peripheral blood mononuclear cells and erythrocytes. Moreover, it has been showed that patients with chronic hepatitis C exhibit an increased production of tumor necrosis factor-alfa (TNF-α), a cytokine that can produce oxidative stress by simulating the generation of reactive oxygen species (ROS) such as superoxide ion (O_2_^.-^) and hydrogen peroxide (H_2_O_2_). ROS can damage cells by causing lipid peroxidation, and oxidative damage of DNA and proteins, and by depleting ATP stores. In the presence of metals (such as Fe ^3+^), O_2_^.- ^can react with H_2_O_2 _to generate a hydroxyl radical than become even more reactive and cytototoxic than O_2_^.- ^or H_2_O_2 _[2].

Lipid peroxidation is caused by free radicals leading to oxidative destruction of polyunsaturated fatty acids constitutive of cellular membranes. Their destruction leads to the production of toxic and reactive aldehyde metabolites such as malondialdehyde (MDA) and 4-hydroxynonenal (HNE). These highly cytotoxic metabolites, produced in relatively large amounts, can diffuse from their site of origin to attack distant targets and form covalent bonds with various molecules. Therefore, recognition of lipid peroxidation is of interest, as the deleterious effects of this process, including fibrogenesis, might be prevented by administration of scavenging systems or antioxidants.

Most previous studies investigating lipid peroxidation dealt with blood and tissue extracts by indirect quantitative methods; the thiobarbituric acid test has been the most commonly applied. Using this procedure, an increase in MDA was observed in the serum, and the liver of patients with chronic hepatitis C. Recently, specific antibodies against MDA and HNE adducts have been raised [3].

Cells are protected against oxidative insults by natural antioxidant products, notably glutathione, and by diverse antioxidant enzymes such as superoxide dismutase (SOD), catalase (CAT) and glutathione peroxidase (GSH-Px) [2]. Oxidative stress develops when the disturbance in balance between the reactive oxygen forms produced in excess, and the factors preventing their harmful effect occur.

Researchers revealed that ROS might be one of the causes of gastrointestinal tract diseases, for example; acute pancreatitis, ulcerative colitis or chronic peptic or duodenal ulcer disease. A significant role of ROS was demonstrated in non-viral hepatitis, especially in hemochromatosis, Wilson disease, and alcoholic lesion of the liver [4]. Although the main role of immunological mechanisms in pathogenesis of chronic viral hepatitis B and C were demonstrated, researchers also concentrate on the problem of oxidative stress in pathology of the diseases.

Many attempts have been made to find serological or cellular markers that help in the clinical management and evaluation of treatment response of chronic hepatitis C (CHC) patients, including plasma viral load, plasma anti-HCV IgM or other soluble immune factors, hepatic viral RNA, etc.; however, repeated biopsies are needed to evaluate disease progression [5]. Although interferon-alfa is still used in treatment of chronic viral hepatitis, its effect on the oxidant and antioxidant status of patients is not well known, which may be significant in deciding on the type on therapy, follow-up and prognosis of patients. The aim of our study is to determine the role of oxidative stress on hepatic damage in the patients with the chronic hepatitis C virus infection and the effect of pegylated interferon alfa-2b plus ribavirin combination therapy on oxidative stress.

## Materials and methods

### Patients

Nineteen patients (11 males and 8 females), mean age 38 ± 12 years, with chronic HCV infection were included in this study. The infection group was selected for this study from out patient clinic unit of the Gülhane Military Medical Academy (GMMA) infectious diseases department. Diagnosis of chronic hepatitis C was based on elevation of serum transaminases, positivity for anti-HCV antibodies (ELISA second generation Ortho Diagnostic Systems, Raritan, NJ), and presence of HCV-RNA (reverse transcription-PCR) in serum and histological evidence of chronic hepatitis. The patients with other chronic or autoimmune liver diseases were excluded from the study.

### Control group

The control group (CG) consisting of 28 healthy individuals (18 males and 10 females), mean age 26.67 ± 6.14 years, were selected from voluntary blood donors of the GMMA. They were selected on the basis of general physical examination. They were seronegative for HCV, HBV, HIV, HBsAg, anti-HBc total, and anti-HCV tests were negative in the control group. No previous history of hepatitis and/or chronic alcoholism was evident. They showed no abnormal laboratory findings including liver function tests. They also showed normal levels of aminotransferase. Informed consent was obtained from each patient, and control group individuals.

### Pegylated interferon alfa-2b and ribavirin treatment

The selected patients above received pegylated interferon alfa-2b, 1.5 μg/kg subcutaneous weekly, and ribavirin 200 mg capsules (dose: <75 kg BW: 2 caps in a.m. & 3 caps in p.m., >75 kg BW: 3 caps in a.m. & 3 caps in p.m.) for 48 weeks. After pegylated interferon alfa-2b and ribavirin therapy, patients showed normalized serum transaminase and negative HCV-RNA. In control and patient groups, serum alanine aminotransferase (ALT) and aspartate aminotransferase (AST) levels, erythrocyte MDA levels, erythrocyte CuZn-SOD and erythrocyte GSH-Px activities were measured. In the patient group, these parameters were measured again after the completion of therapy.

### Laboratory method

Blood samples were drawn from the antecubital vein following an overnight fast, by venipuncture into tubes containing EDTA. They were centrifuged for 10 min. at 4000 g and 4°C. After separation of the plasma, the buffy coat was removed and the packed cells washed three times with two volumes of isotonic saline. Then, a known volume of erythrocytes was lysed with cold distilled water (1:4), stored in a refrigerator at 4°C for 15 min. and the cell debris were removed by centrifugation (2000 g at 4°C for 10 min.). The erythrocyte lysates were stored at -70°C until assayed.

CuZn-SOD and GSH-Px activities were measured in the erythrocyte lysates on a UV-VIS Recording Spectrophotometer (UV-2100S, Shimadzu Co., Kyoto, Japan).

Erythrocyte CuZn-SOD activity was measured as previously described by Aydin et al. [6]. Briefly, the erythrocyte lysates were diluted 400-fold with 10 mM phosphate buffer, pH 7.00. 25-μl aliquots were mixed with 850 μl of substrate solution containing 0.05 mmol/L xanthine sodium and 0.025 mmol/L 2-(4-iodophenyl)-3-(4-nitrophenol)-5-phenyltetrazolium chloride (INT) in a buffer solution containing 50 mmol/L CAPS (3-(cyclohexylaminol)-1-propanesulfonic acid) and 0.094 mmol/L EDTA (pH 10.2). 125 μl xanthine oxidase (80 U/L) was added to the mixture, and the increase of absorbance was followed at 505 nm for 3 min. CuZn-SOD activity is expressed in U/ml.

Erythrocyte GSH-Px activity was measured as previously described by Aydin et al. [6]. Briefly, a reaction mixture containing 1 mmol/L Na2EDTA, 2 mmol/L reduced glutathione, 0.2 mmol/L NADPH, 4 mmol/L sodium azide and 1000 U glutathione reductase in 50 mmol/L TRIS buffer (pH 7.6) was prepared. 20 μl of erythrocyte lysate and 980 μl of the reaction mixture were mixed and incubated for 5 min at 37°C. The reaction was initiated by adding 8.8 mmol/L hydrogen peroxide and the decrease of absorbance recorded at 340 nm for 3 min. GSH-Px activity is expressed in U/ml.

Lipid peroxidation was estimated by measurement of thiobarbituric acid reactive substances (TBARS) in erythrocyte lysates by the method previously described by Aydin et al. [6]. After the reaction of MDA with thiobarbituric acid, the reaction product was followed spectrophotometrically at 532 nm, using tetrametoxypropane as a standard. The results are expressed as nmol/ml.

### Statistical analysis

Statistical analyses were done by SPSS (Statistical Package for the Social Sciences Program) 10.01 for Windows statistical program. *Independent-Sample T- test *was used for the comparison of the CHC patients and the control group. *Paired-Simple T*-*test *was used for comparison of CHC patients before and after the 48 weeks of pegylated interferon alfa-2b, and ribavirin treatment. A value of two-sided *p *< 0.05 was considered statistically significant.

## Results

The results obtained show that the serum MDA is significantly increased in selected group of CHC patients (n:19), before pegylated interferon alfa-2b, and ribavirin treatment when compared with healthy subjects (n:28) [(9.28 ± 1.61 vs. 4.20 ± 1.47 nmol/ml, *p *< 0.001)] Table 1. These results show that the patients with CHC are under the influence of increased oxidative stress.

**Table 1:** MDA, SOD, GSH-Px concentration and ALT, AST levels in the CHC patients before the treatment and the control group. (Mean ± Standard Deviation)

	Control group (n:28)	CHC Patients (n:19) Before Treatment	*P*-value
MDA	4,20 ± 1,47	9,28 ± 1,61	<0.001
CuZn-SOD	285,78 ± 96,46	213,84 ± 71,61	<0.05
GSH-Px	8,01 ± 1,79	6,52 ± 1,86	<0.05
ALT	21,53 ± 6,02	95.84 ± 22.68	<0.001
AST	22,50 ± 4,91	80.52 ± 19.27	<0.001

Values are shown as means, *p *values (<0.05) were derived by the *Independent Samples T-test*. Units were expressed as follows: MDA as nmol/ml; CuZn-SOD as U/ml,; GSH-Px as U/ml; ALT, AST as U/L.

After 48 months treatment of all the CHC patients had eliminated HCV-RNA and normalized serum transaminase. MDA concentration decreased significantly (*p *< 0.001) after pegylated interferon alfa-2b and ribavirin treatment as well as ALT, AST activity in erythrocytes of these patients (Table 2).

**Table 2:** MDA, SOD, GSH-Px concentration and ALT, AST levels in CHC patients before and after the treatment. (Mean ± Standard Deviation)

	CHC Patients Before Treatment	CHC Patients After treatment	*P*-value
MDA	9,28 ± 1,61	4,88 ± 1,22	<0.001
CuZn-SOD	213,84 ± 71,61	357,94 ± 82,10	<0.001
GSH-Px	6,52 ± 1,86	9,47 ± 1,82	<0.001
ALT	95.84 ± 22.68	26,73 ± 10,65	<0.001
AST	80.52 ± 19.27	25,52 ± 8,68	<0.001

Values are shown as means, *p *values (<0.05) were derived by the *Paired-Simple T-test Independent Samples T-test*. Units were expressed as follows: MDA as nmol/ml; CuZn-SOD as U/ml,; GSH-Px as U/ml; ALT, AST as U/L.

Treatment with pegylated interferon alfa-2b, 1.5 μg/kg subcutaneous weekly and ribavirin 200 mg capsules (dose: <75 kg BW: 2 caps in a.m. & 3 caps in p.m., >75 kg BW: 3 caps in a.m. & 3 caps in p.m.) during 48 weeks, led those patients whose ALT, AST activity normalized in serum, to a concomitant decrease of the erythrocytes' MDA content.

We compared the average superoxide dismutase level in erythrocytes of patients with CHC before treatment (213.84 ± 71.61 U/ml) with the average SOD activity in control group (285.78 ± 96.46 U/ml) (p < 0.001). We demonstrated that average SOD activity in patients with CHC is significantly statistically lower (p < 0.05) than in healthy control group.

The average glutathione peroxidase activity in erythrocytes of patients with CHC before treatment were 6.52 ± 1.86 U/ml. It was significantly statistically lower (p < 0.05) than average activity of GSH-Px in erythrocytes of healthy control group (8.01 ± 1.79 U/ml). Table 1 shows the activities of antioxidant enzymes in erythrocyte from the CHC before treatment for patients, and for the control group.

These findings in CHC patients before the treatment demonstrated that antioxidant status was compromised with several important components of the antioxidant defense mechanism being significantly decreased.

## Discussion

The reports from several studies have produced clear evidence that there exists a good correlation between type and severity of disease and antioxidant level in the blood [7]. Although the main role of the immunological mechanisms in pathogenesis of the chronic viral hepatitis B and C was demonstrated, researchers also concentrate on the problem of oxidative stress in the pathology of the diseases. Oxidative stress develops when the disturbances between reactive oxygen forms are produced in excess and the factors preventing their harmful effect occur. Enzymatic antioxidant defense of the organism includes: SOD, CAT, and GSH-Px. Superoxide dismutase protects a cell from toxic effect of superoxide radicals as it catalyzes the dismutation reaction of the radicals [1]. Glutathione peroxidase decomposes hydrogen peroxide but it also converts lipid peroxides to harmless molecules protecting the cells from the consequences of lipid peroxidation. GSH-Px removes H_2_O_2 _by the oxidation of reduced glutathione. Oxidized glutathione (GSSG) is produced and it is reduced again by glutathione reductase, and the NADPH (produced in pentose cycle) [4].

Oxidative stress has been detected in almost all clinical and experimental conditions of the chronic liver diseases [8]. There are many studies about the oxidant stress in chronic hepatitis C patients. De Maria et al. [9] showed that MDA, a product of polyunsaturated fatty acid peroxidation, was elevated in the liver and the blood. Paradis et al. [3] also demonstrated MDA-protein adducts immunohistochemically in infected liver tissue. Boya et al. showed that the peripheral blood mononuclear cells from patient of chronic hepatitis C had increased MDA concentrations, and enhanced SOD activity. MDA is reflection of lipid peroxidation and SOD is an important antioxidant defense enzyme that converts superoxide into hydrogen peroxide. Increased SOD activity appears to be an adaptive response to increased generation of the superoxide ions [1]. Romero et al. showed higher serum malondialdehyde values in chronic hepatitis C patients than healthy subjects before the interferon treatment [5].

Our results showed that serum MDA is significantly increased in CHC patients before the treatment when compared with healthy subjects (*p *< 0.001). The results presented confirm the involvement of the oxidative stress as a part of pathophysiology of CHC. Thus, our findings support the existence of the oxidative stress in patients with chronic HCV infection and are in agreement with the studies mentioned above.

In our study MDA concentration decreased significantly (*p *< 0.001) after pegylated interferon alfa-2b, and ribavirin treatment as well as ALT, AST activity, in erythrocytes of these patients. Romero et al. also demonstrated significantly lower mean value of serum MDA levels after the interferon treatment compared with the pre-treatment levels [5]. Higueras et al. showed decrease in serum TBARS content after treatment with 5 MU interferon, three times a week during two months of chronic hepatitis C patients [10]. We suggest the routine use of MDA assay as additional relevant information for the clinical management of this disease. The results presented confirm the value of MDA as laboratory test in the management of liver diseases and as a useful tool for observing the pathogenic and/or the therapeutic mechanism of this viral infection.

Glutathione (GSH), the most abundant non-enzymatic antioxidant present in the cell, plays an important role in the defense against oxidative-stress-induced cell injury. In the cells glutathione is present mainly in its reduced form. Reduced GSH can be converted to oxidized glutathione (GSSG) with the GSH-Px, which is revertible to the reduced form with the glutathione reductase (GR). Cells are also equipped with the enzymatic antioxidant mechanisms that play an important role in the elimination of free radicals [1].

Suarez et al. studied the group of 100 individuals with chronic hepatitis C. He demonstrated GSH decrease in the blood serum of patients who were not treated with interferon. In contrast, GSH concentration was higher in the blood of patients responding satisfactorily to the treatment. The author set forth a theory explaining the cytopathic effect of a virus causing glutathione deficiency. Kramer et al. studied antioxidant enzymes and they found diminished GSH-Px level in the blood serum and the erythrocytes, in patients with abnormal liver function [4]. Pak et al. also demonstrated low activities of GSH-Px and SOD in adults with acute hepatitis B [11].

Results of our study are consistent with the reports by Suarez and associates. In the current study we demonstrated that glutathione peroxidase level and superoxide dismutase level are decreased in erythrocytes of patients with chronic hepatitis C. It probably decreased the antioxidant barrier efficiency in studied CHC patients. When the activity of the enzymes mentioned above is insufficient, an organism is not capable to neutralize free oxygen radicals that are produced in excess. It leads in consequence to hepatocyte lesion [4].

Chrobot et al. demonstrated that SOD and catalase levels decreased both in group of children with chronic hepatitis C, and B. Kramer and associates studied the small group of children with abnormal function of liver cell, and demonstrated decreased activity of SOD in comparison to the healthy children [4]. Loginow studied antioxidant system in adults with chronic active hepatitis, and demonstrated SOD decrease correlating with severity of inflammatory process [12]. Study by Yasuyama et al. showed decrease of SOD levels in liver tissue of patients with acute, and chronic hepatitis accompanied by fatty degeneration while comparing with patients with the liver inflammatory diseases of different etiology [13]. In another study Irshad et al. found SOD activity significantly low in the chronic active hepatitis C patients [7]. On the contrary, some author has detected high SOD levels in CHC patients [1,2].

Similarly, our results showed that SOD was significantly decreased in CHC patients before treatment when compared with healthy subjects (*p *< 0.001). This result may indicate decreased antioxidant capacity in chronic hepatitis C patients.

The reduction in the amount of SOD, and GSH-Px reflects both a decrease in the synthesize capacity of liver, and the antioxidant defense power of the patients with CHC. It can be argued that increased lipid peroxidation is caused by the inflammation related to viral infection and decreased the antioxidant levels may be an early marker of the oxidative stress. Lipid peroxides formed can be chemotactic for the neutrophils causing increased inflammation, which further drives oxidant-mediated injury in the liver [14].

In the viral hepatitis, virus also infects the peripheral lymphocytes. The infected lymphocytes produce interferon to stimulate healthy cells against viruses [15]. The pathogenetic mechanisms through which HCV causes cell damage remain obscure, although it has been suggested that the oxidative stress may play a pathogenetic role in this infection. The patients with chronic hepatitis C exhibit an increased production of TNF-α, a cytokine that can produce oxidative stress by stimulating the generation of oxygen ROS [2]. Although interferon and ribavirin are not antioxidants, their antiviral capacity might reduce viral load and inflammation and perhaps though this mechanism might reduce virus-induced oxidative stress.

Several clinical trials have previously suggested a beneficial effect of antioxidants in patients with chronic HCV infections. Intravenous administration of glycyrrhizin, a free radical scavenger, decreases elevated plasma transaminase enzymes, and improves histology in patients with chronic HCV infections [16,17]. A combination of three potent antioxidants (alpha-lipoic acid, silymarin, and selenium) induced marked clinical, laboratory and histologic improvement in chronic HCV patients [18,19]. The results of the study by Melhem et al. suggest that antioxidative treatment using a combination of multiple antioxidants in patients with chronic HCV infections, may ameliorate the inflammatory response as measured by liver enzymes and liver biopsy inflammatory score [20]. Herbay et al. [21] observed that high vitamin E supplementation improves the aminotransferase status in patients who have chronic HCV. Murakami et al. demonstrated that antioxidant vitamin (E and C) supplementation during interferon alfa-2b prevented decrease in eicosapentaenoic acid of mononuclear cell phospholipids. It might be possible to enhance the efficacy of combination therapy of interferon alfa-2b and ribavirin [22]. In contrast, Look et al. showed that adjuvant antioxidative therapy by N-acetylcysteine/Selenium co-supplementation did not increase the antiviral response to six-month interferon alfa monotherapy in chronic hepatitis C patients [23].

As a conclusion, lower pretreatment levels of antioxidants and higher level MDA level might be probable marker of the oxidative stress. Reversal change of these levels with completion of the treatment may indicate a correlation between the oxidative stress and the viral pathogenesis. Antioxidant supplement can be added in these patients during their clinical survey. However, further investigations to highlight this issue are recommended.

## Note

Chronic Hepatitis C patients after pegylated interferon alfa-2b and ribavirin therapy showed values of SOD, GSH-Px were significantly higher than pretreatment levels (Table 2). MDA levels of patients with CHC were significantly reduced while SOD and GSH-Px levels increased after treatment. ALT, AST levels of the same group were also reduced to the control levels after treatment.
